# Glutamatergic medications as adjunctive therapy for moderate to severe obsessive-compulsive disorder in adults: a systematic review and meta-analysis

**DOI:** 10.1186/s40360-021-00534-6

**Published:** 2021-11-04

**Authors:** Fatemeh Hadi, Shayan Kashefinejad, Leila Kamalzadeh, Saba Hoobehfekr, Mohammadreza Shalbafan

**Affiliations:** 1 School of Medicine, Shahid Beheshti University of Medical Sciences, Tehran, Iran; 2 Mental Health Research Center, Psychosocial Health Research Institute, Department of Psychiatry, School of Medicine, Iran University of Medical Sciences, Tehran, Iran; 3 Roozbeh Psychiatric Hospital, Tehran University of Medical Sciences, Tehran, Iran; 4 Brain and Cognition Clinic, Institute for Cognitive Sciences Studies, Tehran, Iran

**Keywords:** Glutamate, Riluzole, Memantine, Minocycline, Obsessive-compulsive disorder, Systematic review

## Abstract

**Background:**

Obsessive-compulsive disorder (OCD) is among the most disabling neuropsychiatric conditions characterized by the presence of repetitive intrusive thoughts, impulses, or images (obsessions) and/or ritualized mental or physical acts (compulsions). Serotonergic medications, particularly Selective Serotonin Reuptake Inhibitors (SSRIs), are the first-line treatments for patients with OCD. Recently, dysregulation of glutamatergic system has been proposed to be involved in the etiology of OCD. We designed this systematic review and meta-analysis to evaluate clinical efficacy of glutamatergic medications in patients with OCD, according to the guidelines of Cochrane collaboration.

**Method:**

We searched Medline, Scopus, and Cochrane library without applying any language filter. Two of the authors independently reviewed search results for irrelevant and duplicate studies and extracted data and assessed methodological quality of the studies. We transformed data into a common rubric and calculated a weighted treatment effect across studies using Review Manager.

**Results:**

We found 476 references in 3 databases, and after exclusion of irrelevant and duplicate studies, 17 studies with total number of 759 patients with OCD were included. In the present review we found evidence for several drugs such as memantine, N-acetylcysteine (NAC), Minocycline, L-carnosine and riluzole. Glutamaterigic drug plus SSRIs were superior to SSRI+ Placebo with regard to Y-BOCS scale [standardized mean difference (SMD = − 3.81 95% CI = − 4.4, − 3.23).

**Conclusion:**

Augmentation of glutamatergic medications with SSRIs are beneficial in obsessive-compulsive patients, no harmful significant differences in any safety outcome were found between the groups.

**Supplementary Information:**

The online version contains supplementary material available at 10.1186/s40360-021-00534-6.

## Background

Obsessive-compulsive disorder (OCD) is among the most disabling neuropsychiatric conditions characterized by the presence of repetitive intrusive thoughts, impulses, or images (obsessions) and/or ritualized mental or physical acts (compulsions) [1]. OCD affects approximately 1–2% of adult general population worldwide [2]. It is associated with significant functional impairment, both due to the primary illness, as well high comorbidity with other psychiatric disorders. Abnormalities in serotonin and/or dopamine neurotransmission have been suggested to underlie the development of OCD [3, 4].

Recommended first-line pharmacotherapies for OCD are serotonergic antidepressants, such as selective serotonin reuptake inhibitors (SSRIs) and clomipramine [5, 6]. However, estimates suggest that around 30–60% of patients do not improve or show a partial response to adequate serotonergic antidepressant treatment, implying that serotonergic dysregulation may not be the one but rather one of many important mechanisms that are involved in the pathophysiology of OCD.

Recently, researchers have proposed that glutamatergic dysfunction, especially in the cortico-striato-thalamo-cortical (CSTC) circuitry may play a key role in the pathophysiology of OCD [7, 8]. Glutamate is the principal excitatory neurotransmitter in the central nervous system. It is also a precursor for gamma-amino butyric acid (GABA), the main inhibitory neurotransmitter in the brain, as well as for the amino acid glutamine and the antioxidant molecule glutathione [7]. Glutamate plays a vital role in various physiological processes including neuronal migration and cell maturation particularly by acting on N-methyl-D-aspartate (NMDA) and α-amino-3-hydroxy-5-methyl-4-isoxazolepropionic acid (AMPA) receptors [9]. Abnormally elevated or reduced glutamate is shown to have adverse effect on cortical migration. The striatum, one of the major components of CSTC circuitry, is the largest group of receptive neurons in the basal ganglia, receiving a large glutamatergic excitatory input from the cortex [10]. Evidently, the striatum is responsible for planning cognitive and motor actions [7]. Aberrant glutamatergic signaling between orbitofrontal cortex (OFC), anterior cingulate cortex (ACC) and striatum have been widely recognized to be associated with the development of OCD [11]. Interestingly, recent evidence has also shown in vivo evidence for glutamatergic control of presynaptic serotonin release in the striatum. It is well known that dysregulation of the striatal serotonergic system is a primary pathology in OCD [12].

The most direct evidence suggesting altered glutamate homeostasis in OCD derived from cerebrospinal fluid (CSF) studies. These early studies demonstrated that glutamate is excessive in the CSF of a subset of untreated patients with OCD [13, 14]. Additional studies using magnetic resonance spectroscopy (MRS) indicated that glutamate and related compounds are elevated in the basal ganglia and reduced in the anterior cingulate cortex in patients with OCD [7, 15]. There is also some evidence to suggest that polymorphisms in glutamate-associated genes may contribute to OCD risk [16]. Among the implicated glutamate-associated genes in OCD, the most consistent candidates are the SLC1A1 which encodes the neuronal glutamate transporter excitatory amino acid transporter 3 (EAAT3), and The SAPAPs (synapse associated protein 90/postsynaptic density-95-associated proteins)/ DLGAPs (disks large-associated proteins) which are key components of the postsynaptic complex that anchors and spatially organizes glutamate receptors [17, 18].

Further to the aforementioned evidence on glutamatergic dysfunction in OCD, the potential benefits of some glutamate-modulating agents such as riluzole, memantine, N-acetylcysteine (NAC), D-cycloserine, and ketamine have been demonstrated in the treatment of OCD [10, 19]. However, few writers have been able to draw on any systematic research into the potential utility of these agents. Hence, this paper will systematically review the research conducted on the clinical efficacy of glutamate-modulating agents in the treatment of patients with OCD, aiming to serve as a base for future studies in this area.

## Methods

We conducted this systematic review in accordance with the Preferred Reporting Items for Systematic Reviews and Meta-Analyses for Protocols guidelines [20].

### Search strategy and selection criteria

In this systematic review and meta-analysis, controlled clinical trials (irrespective of blinding and randomization) investigating clinical efficacy of glutamatergic drugs (irrespective of modes of administration, dosage, frequency and duration) in patients with OCD (irrespective of age, gender or race) were included (Appendix 1).

The primary search process was conducted in Web of Science, PubMed, Scopus, ScienceDirect, Cochrane library and Google Scholar databases based on the search strategies described in the protocol (Appendix 1) to gather the body of evidence available from original articles published up to 2021 in English. The first author conducted an electronic database search. Then, the titles and abstracts of studies initially selected were screened to eliminate duplicate citations and those that were obviously irrelevant. The full texts of the remaining studies were obtained for quality assessment, data collection and analysis.

### Data extraction and quality assessment

After the initial screening, the full texts were reviewed by two independent researchers to include eligible articles according to the inclusion criteria.

Detailed data extraction was performed based on the pre-designed data extraction forms. Extracted information included the study design, name and address of the corresponding author, participants’ characteristics, interventions and outcomes. The methodological quality of the included studies was then evaluated according to the Cochrane risk of bias assessment tool [21]. In case of disagreement between authors, opinion was sought from a third author.

### Evidence synthesis

We developed an evidence synthesis of the findings of the included studies using systematic approaches such as textual descriptions, tabulation, and transforming data into a common rubric using Review Manager (Version 5.3. Copenhagen: The Nordic Cochrane Centre, the Cochrane Collaboration, 2014). Missing data were handled using sophisticated statistical analysis techniques.

A meta-analysis was performed and the weighted average treatment effect was estimated. Heterogeneity across studies were evaluated by the chi-square statistic and calculation of I^2^(defined as I^2^ > 40% and/or chi-square statistic *p* < 0.1). A random-effects model was used in the case of statistical heterogeneity. Moreover, we applied a subgroup analysis in case of clinical heterogeneity.

## Results

### Description of included studies

**Fig. 1:**
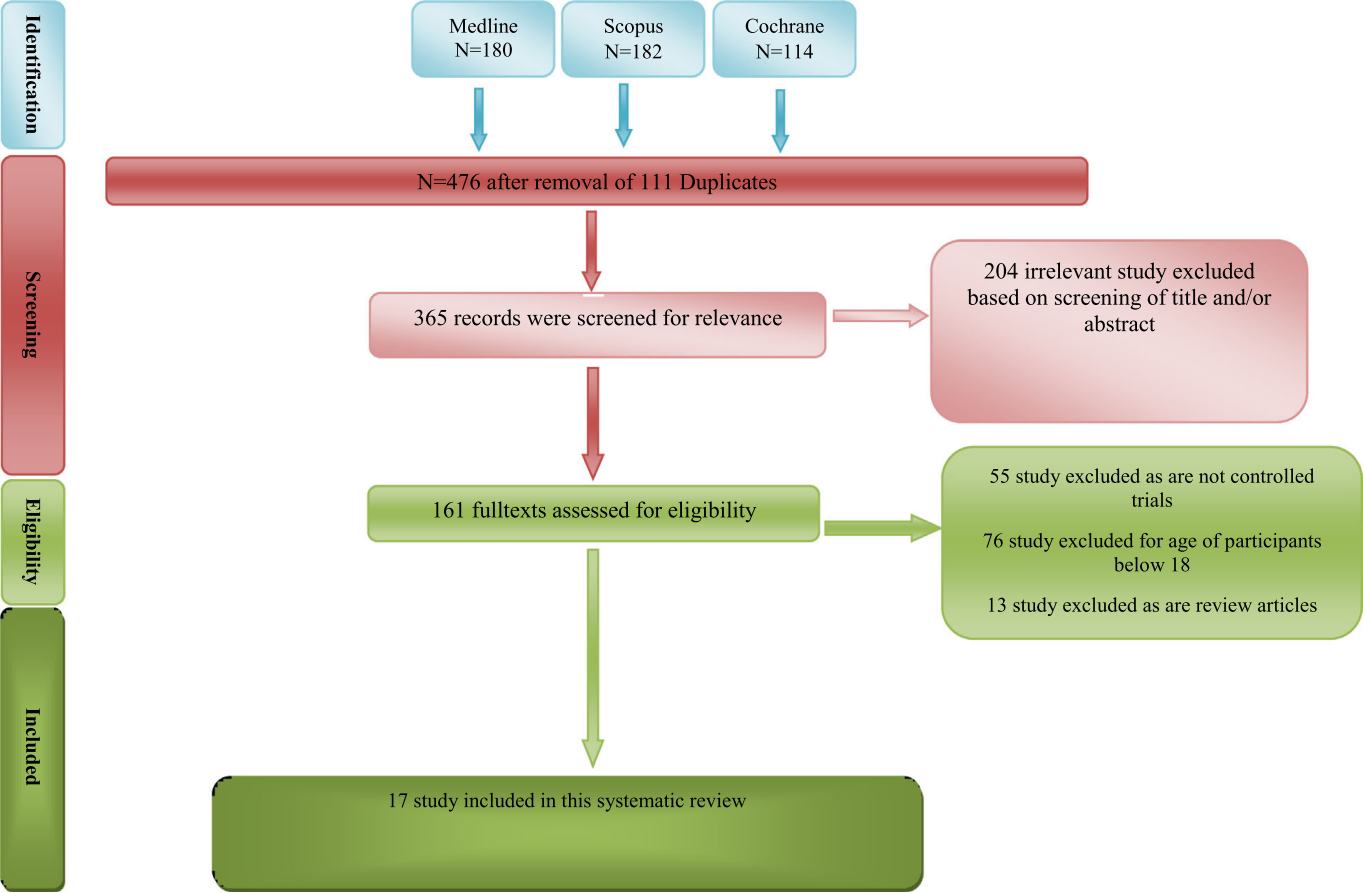
Flowchart of study selection for systematic review and meta-analysis of Glutamatergic Medications as Adjunctive Therapy for Moderate to Severe Obsessive-Compulsive Disorder in Adults

We found 476 studies of interest in the initial electronic searches. We then excluded 111 duplicate citations using Endnote software and 204 articles due to obvious irrelevancy of their topics in primary screening (Fig. 1). In secondary screening of 161 full texts, we excluded 144 articles, and finally included 17 controlled trials with 759 patients with OCD in this systematic review (Table 1).

**Table 1:** Controlled clinical trials reporting Glutamatergic drugs in OCD

	Study (first author, year of study)	Study patients and main groups	Interventions	Outcomes, results and relationships
1	Afshar et al., 2012	**Patients with refractory OCD**(*n* = 38) 1- **control group** (*n* = 24) 2- **experimental group** (*n =* 24)	1- **Control group: placebo, identical pills** 2- **Experimental group**: initial dosage of 600 mg/d of NAC, which doubled weekly to a maximum dose of 2400 mg/d • Serotonin reuptake inhibitor treatment continued throughout the study with the same dose as the preintervention phase • 12 wks follow up	➢ Y-BOCS: Mean difference 95%CI; − 5.14 [− 6.87, − 3.41] ➢ Adverse effects: no unusual or serious adverse event was observed. The adverse events reported during trial were only gastrointestinal. Eight patients in the NAC group reported nausea/vomiting of mild to moderate intensity compared with 2 patients in the placebo group. Mild diarrhea was reported by 4 patients in the NAC group but none of the patients in the placebo group
2	Arabzadeh et al., 2017	**Those with a diagnosis of moderate to severe OCD defined by a Yale-Brown Obsessive Compulsive Scale (Y-BOCS) score of ≥ 21 (Goodman, Price, Rasmussen, et al., 1989) were included**(*n* = 50) 1- **control group** (*n =* 25) 2- **experimental group** (*n* = 25) Hepatectomy and radio-chemotherapy were front-line therapies	1- **Control group**: **Fluvoxamine (200 mg) + placebo** 2- **Experimental group: Fluvoxamine (200 mg daily) + L-carnosine 500 mg twice per day** • Follow up after 10 wks	➢ Y-BOCS: Mean difference 95%CI; − 3.00 [− 4.70, − 1.30] *P* value:0.01 ➢ Adverse effects: Headache 6 Dry mouth 6 Nausea 3 Constipation 6 Sweating Frequency of side effects was not different between the two groups.
3	Costa et al., 2017	**Patients with OCD** (*n* = 40) 1- **control group** (*n* = 22) 2- **experimental group** (*n =* 18)	1- **Control group**: **SRI + Placebo** 2- **Experimental group: SRI + N-Acetyl Cysteine** • 16 wks follow up • During the first week, patients started the trial with either NAC 1200 mg (one 600-mg capsule twice a day) or an equivalent number of placebo capsules. In the second week, the dosage was increased to 4 capsules per day (NAC 2400 mg [2 capsules twice a day] or an equivalent number of placebo capsules). Finally, on the third week, the target dose of 5 capsules per day was reached (NAC 3000 mg [2 capsules in the morning and 3 in the evening] or an equivalent number of placebo capsules) and sustained for the remainder of the study.	➢ Overall survival in 8 years* (HR; 95%CI; *P* value**):** 0.69 (0.41–1.15); *P* = 0.1545 ➢ Recurrence-free survival in 5 years* (HR; 95%CI; *P* value**):** 0.31 (0.09–1.07); *P* = 0.0639 ➢ **Toxicity grade 3–4:** none was reported
4	Emamzadehfard et al. 2016	**Patients with OCD** (*n* = 54) 1- **control group** (*n* = 27) 2- **experimental group** (*n =* 27) according to the DSMIV-TR and a Yale–Brown Obsessive Compulsive Scale (Y-BOCS) score of ≥21	1. **Control group**: **fluvoxamine 200 mg/day + placebo** 2. **Experimental group: fluvoxamine 200 mg/day + riluzole 50 mg BD 10 wks follow up**	➢ Y-BOCS: Mean difference 95%CI; − 3.56 [− 6.89, − 0.23] *p* value:0.04 Adverse effects: Experimental group: Drowsiness, 6 (24%) Constipation, 5 (20%) 5 (20%) Dizziness, 4 (16%)Abdominal pain, n (%) 5 (20%) 4(16%) Increased appetite, n (%) 4 (16%) 5 (20%) Decreased appetite, n (%) 3 (12%) 4 (16%) Nausea, n (%) 6 (24%) 5 (20%) Headache, n (%) 4 (16%) 4 (16%) Dry mouth, n (%) 3 (12%) 3 (12%) Cough, 4 (16%) Diarrhea, 6 (24%) 4 (16%) Increase in liver-function tests (4%) 0
5	Esalatmanesh et al., 2016	**Patients with OCD Y-BOCS > 21** (*n* = 132) 1- **control group** (*n* = 66) 2- **experimental group** (*n =* 66) • Almost half of the patients had hepatitis B • Hepatectomy or TACE were frontline therapies	1- **Control group**: **Fluvoxamine 200 mg/day + placebo (identical, same shaped capsule)** 2- **Experimental group: Fluvoxamine 200 mg/day + Minocycline 100 mg twice daily** 10 wks follow up	➢ Y-BOCS: Mean difference 95%CI; −3.21 [− 0.84– − 5.58] *p* value:0.008 ➢ Adverse effects: No difference between to gropus
6	Farnia et al., 2018	**Patients with OCD** (*n* = 99) 1- **control group** (*n =* 33) 2- **experimental group 1** (*n* = 33) 3- **experimental group 2** (*n =* 33)	1. **Control group**: **Fluoxetine 40 mg/day + placebo (identical pills)** 2. **Experimental group 1: Fluoxetine 40 mg/day + gabapentin 300 mg/day** 3. **Experimental group 2: Fluoxetine 40 mg/day + memantine 10 mg/day** • From baseline to the end of the fourth week, daily dosage of fluoxetine was 20 mg; from the beginning of the fifth week to the end of the eighth week, daily dosage of fluoxetine was 40 mg. • From baseline to the end of the fourth week, daily dosage of gabapentin was 100 mg; fromthe beginning ofthe fifthweek to theend of the eighth week, daily dosage of gabapentin was 300 mg • memantine was given daily at 5 mg in the morning/evening for the four first weeks of the study, and then increased to 10 mg daily for weeks five to eight. Likewise, placebos were given daily in the morning/evening	➢ **memantine vs. placebo:** Mean difference 95% CI: - 1.12 [− 0.916, − 3.1622] *P*-value:0.2 ➢ **Gabapentin vs. placebo** Mean difference 95% CI: - 2.550[− 0.7895, − 4.3105] *P-*value:0.0061 ➢ Adverse effects: More rash was seen in memantine group than control group Sleepiness was reported in gabapentin group
7	Ghaleiha et al., 2013	**Patients with OCD** (*n* = 42) • **control group** (*n* = 21) • **experimental group** (*n =* 21)	1- **Control group**: **fluvoxamine 200 mg/day + placebo (with the same shape and taste as memantine)** 2- **Experimental group: fluvoxamine 200 mg/day + memantine 20 mg/day** • All patients received fluvoxamine100mg/ day for the first four weeks of the trial followed by 200 mg/day for the rest of the study • 8 wks follow up	• Y-BOCS: has not reported the mean of score after the trial. Just reports remission rate • Adverse effects in control group: Drowsiness (26%) Headache (21%) Constipation (31%) Dizziness (21%) Fatigue (16%) Nausea (21%) Decreased appetite (26%) Itching (10%) Nervousness (21%) Rash (5%) in experimental group: Drowsiness (21%) Headache (16%) Constipation (31%) Dizziness 3 (16%) Fatigue (16%) Nausea (26%) Decreased appetite (21%) Itching (16%)Nervousness (21%) Rash (10%)
8	Greenberg et al., 2009	**Patients with OCD** (*n =* 24) • **control group** (*n* = 12) • **experimental group** (*n =* 12)	1. **Control group**: **placebo fluid (dextrose, fructose, fine granular citric acid, orange flavoring and ProSweetTM flavor enhancer)** 2. **Experimental group: glycine powder 30 g dissolved in water or juice, twice daily** • The experimental intervention was adjunctive to participants’ continuing psychotropic and psychotherapeutic regimens, which were managed by their treatment providers in the community. • 12 wks follow up	➢ Y-BOCS: Mean difference 95%CI; −5.30 [− 11.56, 0.96];*p* value: 0.1650 ➢ Adverse effects: constipation 1; nausea or disagreeable taste 8 out of 16 dropped patients
9	Haghighi et al., 2013	**Patients with OCD** (*n =* 40) • **control group** (*n* = 20) • **experimental group** (*n =* 20)	1- **Control group**: **placebo (identical pill)** 2- **Experimental group: memantine (5–10 mg/day)** • All patients use medication 1 week prior to the beginning of the study (and continued throughout the study) of an SSRI (e.g., escitalopram, 10 mg/day; citalopram, 30–50 mg/day) or clomipramine (100–175 mg/day) at therapeutic dosages for at least 12 consecutive weeks • 12 wks follow up	➢ Y-BOCS: Mean difference 95%CI; −3.21 [− 0.84– − 5.58] *p* value:0.008 ➢ Adverse effects: light-headedness and vertigo 5% (similar to placebo group)
10	Modarresi et al., 2018	**Patients with OCD** (*n* = 32) • **control group** (*n* = 16) • **experimental group** (*n =* 16)	1- **Control group**: **placebo (identical pills)** 2- **Experimental group: memantine 10 mg/day** • Patients continue their SRI therapy during the study • 12 wks follow up	➢ Y-BOCS: Mean difference 95%CI; −13.53 [− 15.59, − 11.47] *p* value< 0.001 ➢ Adverse effects: Headache 13.3%Constipation6.6% Nausea 6.6% Dizziness 13.3% Decreased appetite 6.6%
11	Mowla et al., 2019	**Patients with OCD** (*n* = 56) • **control group** (*n* = 28) • **experimental group** (*n =* 28) • the patients in this study had failed to respond to at least 12 weeks of treatment with an adequate and stable dose of sertraline, as reflected by a baseline Yale–Brown Obsessive Compulsive Scale (YBOCS) of 18 or greater before enrollment in our trial	1- **Control group**:**sertraline + placebo (identical pills** 2- **Experimental group: Sertraline + pregabalin (75 mg/day initialy, increase 75 mg weekly** • The sertraline dosage had been tittered up until patient’s intolerance. • No pregabalin dose escalation was administered in the case of patient’s intolerance or clinical response.	➢ Y-BOCS: Mean difference 95%CI; −8.82 [− 11.17, − 6.47] *p* value< 0.001 ➢ Adverse effects: Dizziness 4%, drowsiness 18%, headache 4%
12	Naderi et al., 2019	**Patients with OCD** (*n* = 106) • **control group** (*n* = 53) • **experimental group** (*n =* 53) • The patients were not allowed to have received any psychotropic medications during the last 6 weeks or to have participated in psychotropic sessions • met the DSM-5 diagnostic criteria for moderate to severe OCD and had a Yale–Brown Obsessive Compulsive Scale (Y-BOCS) score of > 21	1- **Control group**: **fluvoxamine (100 mg twice a day) + placebo** 2- **Experimental group: fluvoxamine (100 mg twice a day) + amantadine (100 mg daily)** • All patients received 100 mg/day fluvoxamine for 28 days, which was followed by 200 mg/day for the rest of the trial, regardless of their treatment groups	➢ Y-BOCS: Mean difference 95%CI; − 2.31 [− 4.58, − 0.04] *p* value:0.047 Adverse effects: Abdominal pain (5.8%) Decreased appetite (3.9%) Increased appetite (3.9%) Insomnia (1.9%) Headache (3.9%) Nervousness (1.9%) Tremor (1.9%) Constipation (3.9%)
13	Paydari et al., 2016	**Patients with OCD** (*n* = 46) • **control group** (*n* = 23) • **experimental group** (*n =* 23) • (DSM-IV TR) criteria of moderate-to-severe OCD and scored ≥21 in Y-BOCS	1- **Control group**: **fluvoxamine (200 mg daily) + placebo (identical pills)** 2- **Experimental group: fluvoxamine (200 mg daily) + NAC (2000 mg daily)** • The NAC initial dosage was 1000 mg/day (500 mg two times a day) for the first week, followed by 2000 mg/day (1000 mg bid) for the subsequent 9 weeks • 10 wks follow up	➢ Y-BOCS: Mean difference 95%CI; − 2.04 [− 4.97, − 0.88] *p* value:0.16 ➢ Adverse effects: Drowsiness, 18% Constipation 23% Dizziness 27% Vomiting27% Nausea27% Headache18% Dry mouth14% Increased blood Pressure14% Diarrhoea18%
14	Pittenger et al., 2015	**Patients with OCD** (*n =* 38) 1- **control group** (*n =* 18) 2- **experimental group** (*n* = 20) • treatment with an SSRI or clomipramine at a stable effective dose for 8 weeks (by patient report) is an item in inclusion criteria	1- **Control group**: **Placebo** 2- **Experimental group: riluzole 50 mg bid** • all subjects began with a 2-week single-blind placebo lead-in phase, followed by 12 weeks of double-blind riluzole or placebo. In posttrial debriefing, no subjects expressed awareness of this initial placebo lead-in phase. Any subjects experiencing a greater than 25% improvement in the Y-BOCS over this 2-week placebo lead-in phase were excluded from randomization. • 12 wks follow up • Low-dose stable neuroleptic augmentation and benzodiazepine use were permitted • Ongoing psychotherapy of 12 weeks duration was permitted	➢ Y-BOCS: Mean difference 95%CI: − 4.5[− 6.5074, − 2.4926] *p* value:0.002 ➢ Adverse effects: Nausea: < 10% in experimental group
15	Rodriguez 2013	**Patients with OCD** (*n* = 38) 1. **control group** (*n* = 18) 2. **experimental group** (*n =* 20) • Patients on average were off all psychotropic medications for 2.9 years	1. **Control group**: **Placebo (saline infusion)** 2. **Experimental group: Ketamine infusion(0.5 mg/kg)** • Cross-over study • One week duration • Include only the first period of study, because of significant carryover effect	➢ Y-BOCS: Mean difference 95%CI − 5.46 [− 13.15, 2.22] *p* value: 0.1868 ➢ Adverse effects: ° During infusion: Dissociation: 93% unusual content of thought:87% elevated mood: 7% ° Post-infusion:dizziness 20% nausea: 13% headache 13% ° All side effects were resolved after 110 min of infusion
16	Rutrick et al., 2017	**Patients with OCD** (*n =* 50) 1. **control group** (*n* = 24) 2. **experimental group** (*n* = 26) • All patients had used SRI for 12wks before baseline and had insufficient response (Y-BOCS score > 16)	1. **Control group**: **Placebo** 2. **Experimental group: Magovlurant 200 mg BiD** • 19 wks follow up. First 4 wks for dose titration and last 3 wks for taper of mavoglurant • During the study patients remained on their SSRI treatment • Patients who were receiving cognitive behavioral therapy (CBT) as a part of their standard care continued to receive this therapy for the duration of the study.	➢ Y-BOCS: Mean difference 95%CI 1.80 [−4.30, 7.90] *p* value: 0.5658 ➢ Adverse effects [placebo(%) vs experimental(%)]: Headache 8 (33.3) 10 (38.5) Insomnia 2 (8.3) 6 (23.1) Dizziness 2 (8.3) 5 (19.2) Nasopharyngitis 4 (16.7) 2 (7.7) Abdominal pain upper 1 (4.2) 2 (7.7) Abnormal dreams 1 (4.2) 2 (7.7) AST increased 1 (4.2) 2 (7.7) Depression 2 (8.3) 1 (3.8) Fatigue 1 (4.2) 2 (7.7) Vertigo 1 (4.2) 2 (7.7)
17	Sarris et al., 2015	**Patients with OCD** (*n =* 50) 1. **control group** (*n =* 24) 2. **experimental group** (*n =* 26) • patients were on either no treatment or a stable treatment regimen for a minimum of four weeks of current treatment and a minimum of 12 weeks if this is their first OCD treatment	1. **Control group**: **Same shaped cellulose capsule** 2. **Experimental group: NAC 3000 mg/day** • 16 wks follow up • NAC was titrated, 1000 mg/day in week 1, 2000 mg/day in week 2	➢ Y-BOCS: Mean difference 95%CI; − 2.04 -0.59 [− 6.31, 5.13] *p* value: 0.8425 ➢ Adverse effects: heartburn 20%

**Fig. 2:**
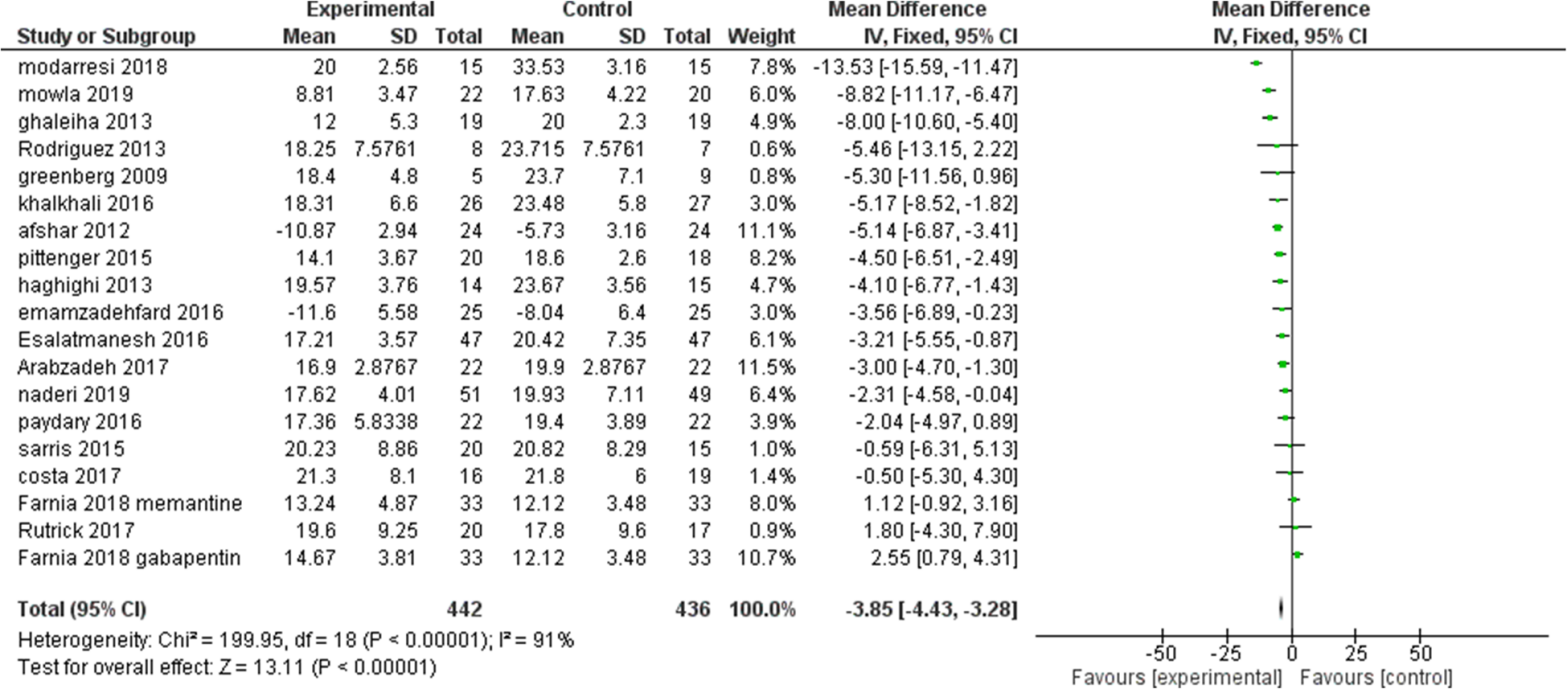
Comparison of Y-BOCS score between patients with moderate to severe OCD groups and control groups in each of the studies and in overall meta-analysis

**Fig. 3:**
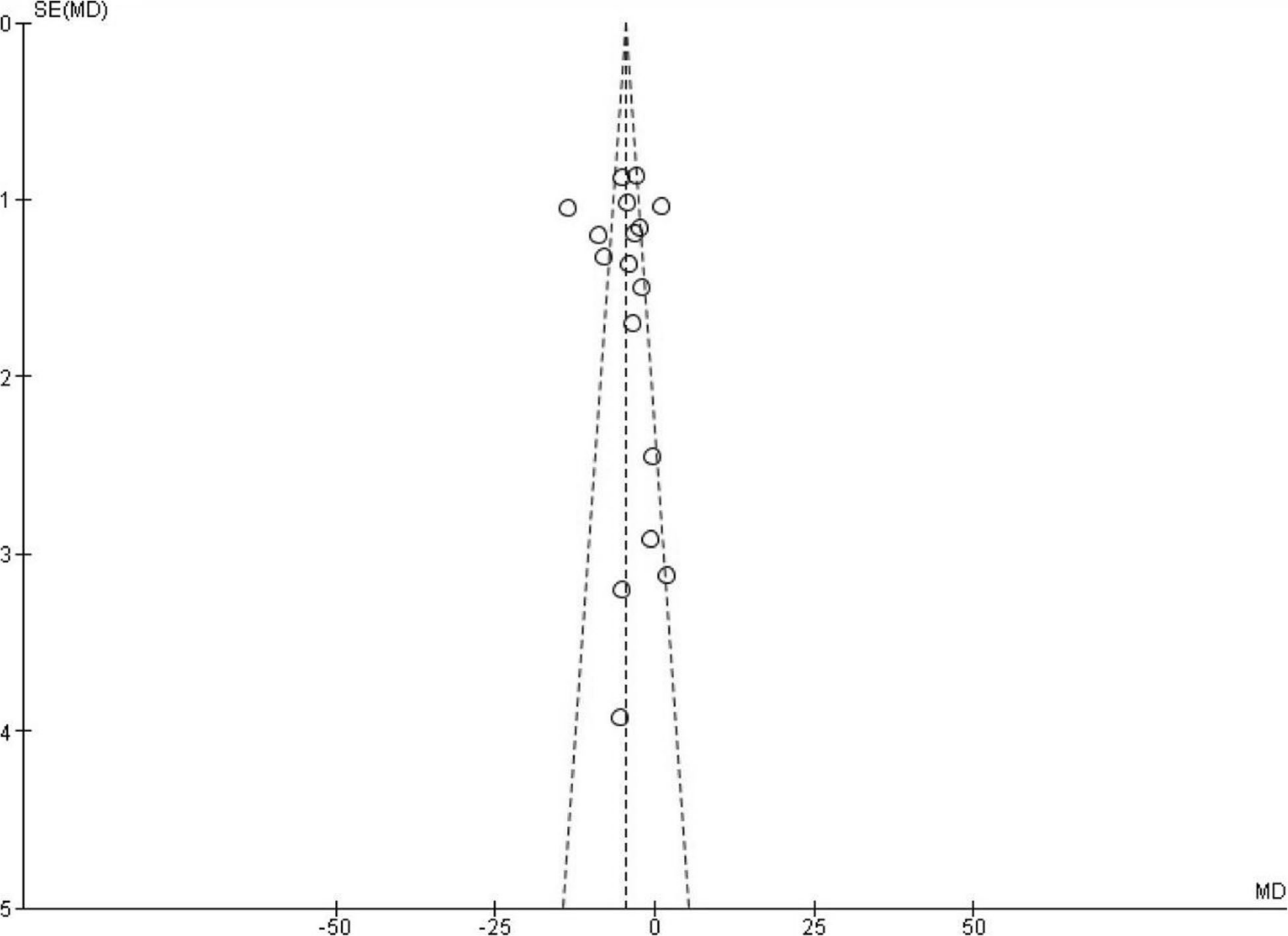
Funnel Plot graph for the studies included in the Meta-Analysis. The asymmetry in the funnel plot may be due to heterogeneity of samples. It means that a subgroup of OCD patients may benefit more from glutamatergic drugs than other groups

Our primary outcome measure was the mean difference in Yale-Brown obsessive-compulsive score (Y-BOCS) ratings before and after pharmacological intervention in experimental and control groups. All except one of the included studies reported Y-BOCS final scores of each group. In the study which these data were not reported, we considered the proportion of treatment responders (as defined by a 35% decline in Y-BOCS scores) in the experimental group compared to the placebo group. A treatment response was significantly more likely in the glutamate-mediating-augmentation group than in the placebo-augmentation group (z = − 3.83, *P* < 0.001). Details of the studies are described in Table 1. Diagram 1 illustrates the forest plot of analyses of the included studies. The results of the assessment of studies for six main biases are shown in Fig. 2. The Funnel plot graph for the studies included is shown in Fig. 3. As can be seen, the overall quality of studies was fair (Table 2).

**Table 2:**
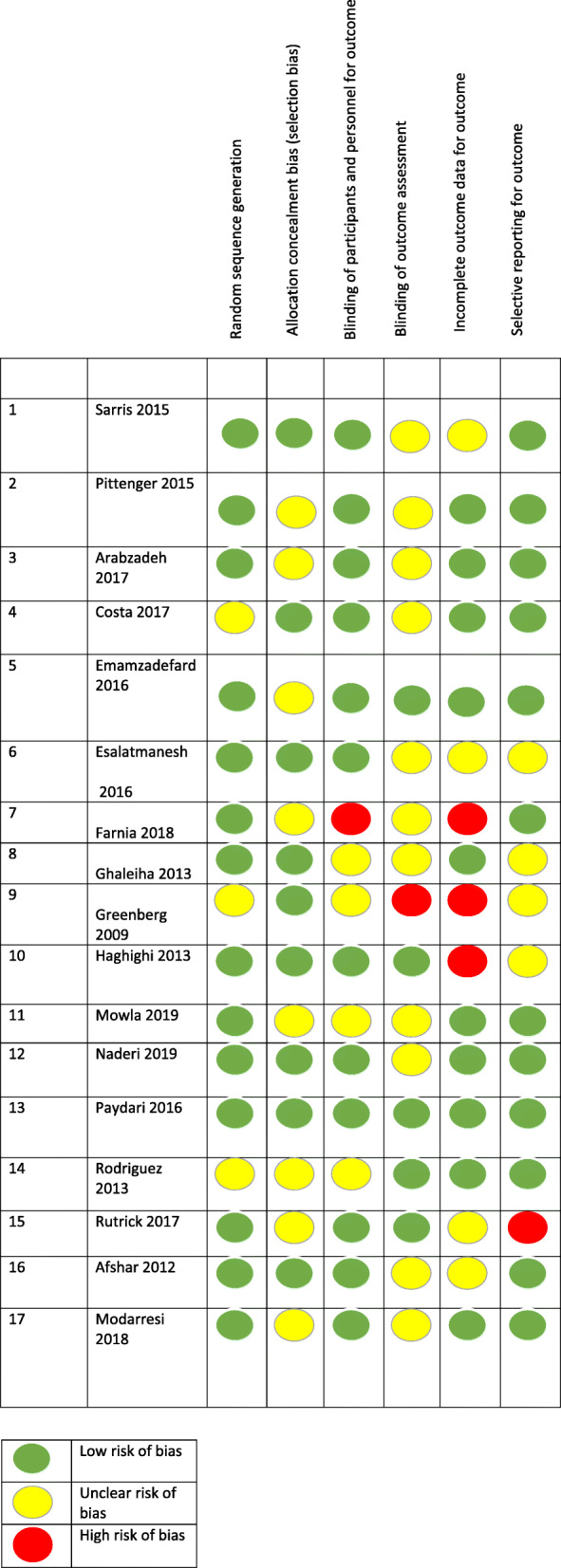
Table of risk of bias for included studies considering Cochrane ‘Risk of bias’ tool. The overall quality of studies was fair

### Memantine

Memantine is one of the approved medications for moderate to severe Alzheimer’s disease. It is a non-competitive antagonist of NMDA receptor, one of the main receptors of glutamatergic system [22]. Memantine blocks the effects of sustained, pathologically elevated levels of glutamate that may otherwise lead to neuronal dysfunction [23].

Four of seventeen studies in this review included memantine as an adjuvant therapy. In three of these studies reporting final Y-BOCS scores, the mean difference of Y-BOCS score between two groups with 95% confidence interval was − 5.68[− 6.96, − 4.41] (*P*-value< 0.0001).

One of the studies only reported the remission rate of the responders (> 35% decrease in Y-BOCS score). In which, 89% of the patients in the memantine group compared with 32% of the patients in the placebo group achieved remission (χ2(1) = 13.328, *P* < 0.001) [24].

### Minocycline

Minocycline is a known glutamatergic agent with therapeutic effects on neurodegenerative diseases which might be achieved through the blockade of glutamate-mediated excitotoxicity. Moreover, this antibiotic is known for its antioxidant and anti-inflammatory characteristics, which could further explain its neuroprotective effects. The beneficial role of minocycline in the treatment of schizophrenia, depressive and autistic symptoms is reported in previous research [25, 26].

In one of the studies, the efficacy of minocycline was assessed as an augmentative agent to fluvoxamine in the treatment of patients with OCD [27]. Significantly lower Y-BOCS scores were achieved in the minocycline group compared to the placebo group at the end of the study (t-score: − 2.84, *P* value: 0.0084).

### L-carnosine

L-carnosine is a nutritional complementary agent with both antioxidant and glutamatergic properties. Carnosine reduces the glutamate levels in the central nervous system via upregulation of the glutamate transporter 1 [28–30]. One of the studies assessed the effect of L-carnosine as adjunct therapy to fluvoxamine in OCD and found significant decrease in final Y-BOCS score (t42:-2.62, *P*-value:< 0.001) [31].

### Riluzole

Riluzole, an anti-glutamatergic agent, is mainly known as a treatment of Amyotrophic Lateral Sclerosis (ALS) [32]. A 10-weeks randomized placebo-controlled trial examined the efficacy of riluzole in the management of OCD. This study showed significant improvement in the patients treated with Riluzole [33]. Two studies were found which used Riluzole for the trial [34]. The reduction of Y-BOCS score at the end of studies were significantly lower than control group. [Z = 4.85 (*P* < 0.00001)].

### N-acetylcysteine (NAC)

NAC is known as a regulator of glutamatergic system and can prevent pre-oxidant effect of glutamate. It has been proposed as a potential therapy for OCD since it can regulate the exchange of glutamate and prevent its pre-oxidant effects [35]. Four studies in this review included this medication. In the analysis of these studies, there was a significant decrease in the Y-BOCS scores in the experimental group [Z = 5.4(P: 0.000139)] [36–39].

## Discussion

Many glutamate-modulating drugs have been reported to have clinical efficacy in the treatment of OCD. As previously mentioned, riluzole which is an anti-glutamate drug, can be effective in the treatment of refractory OCD. This therapeutic effect can be considered an evidence for the abnormal elevation of glutamate in the CNS of patients with OCD [40, 41].

Based on the findings of this review, memantine has strong evidence supporting its clinical efficacy in the treatment of OCD which is in agreement with previous reviews [42]. Additionally, there is promising evidence on the therapeutic effects of riluzole and NAC in other studies [43, 44]. There is also confirmative data on the potential utility of minocycline by Marinova et al. [19]. Other medications with different mechanisms have also been proposed to be effective in the treatment of OCD. For instance, topiramate (through gamma-Aminobutyric acid (GABA) and AMPA/kainite-type glutamate receptors) [45] and lamotrigine (through GABA and reduction of the presynaptic release of glutamate [46], have shown some efficacy in patients with OCD. Moreover, D-cycloserine, a partial agonist of NMDA receptor, has demonstrated some supporting evidence [10, 15, 34, 42, 44, 45], but we did not find any eligible study on these drugs. However, it is unfortunate that this study did not include data reported in peer-reviewed publications other than journal articles. Therefore, it is important to bear in mind the possibility of publication bias.

## Conclusions

In summary, there is supporting evidence for glutamate-modulating drugs in treating moderate to severe OCD as an alternative or adjunctive therapy. In the present review we found evidence for several drugs such as memantine, NAC, minocycline, L-carnosine and riluzole. Further research is needed to determine neuroanatomical, neurochemical and basic genetics for the new line of treatments in OCD. The efficacy, effectiveness and risks associated with these glutamate- modulating drugs for the treatment of moderate to severe OCD should be further investigated. In future investigations, it would also be interesting to identify and analyze possible moderator variables.

## Supplementary Information

**Additional file 1.**

## Data Availability

The datasets used and analyzed during the current study are available from the corresponding author on reasonable request.

## Abbreviations

OCD: Obsessive-compulsive disorder
SSRIs: Selective Serotonin Reuptake Inhibitors
CSTC: Cortico-striato-thalamo-cortical
CNS: Central Nervous System
ASD: Autism Spectrum Disorder
MDD: Major Depressive Disorder
GABA: Gamma-Aminobutyric acid
NMDA: N-methyl-d-aspartate
AMPA: α-amino-3-hydroxy-5-methyl-4-isoxazolepropionic acid
OFC: Orbitofrontal cortex
ACC: Anterior Cingulate Cortex
CSF: Cerebrospinal fluid
MRS: Magnetic Resonance Spectroscopy
EAAT3: Excitatory amino acid transporter 3
SAPAPs: Synapse associated protein 90/postsynaptic density-95-associated proteins
DLGAPs: Disks large-associated proteins
NAC: N-acetylcysteine
Y-BOCS: Yale-Brown obsessive-compulsive score
ALS: Amyotrophic Lateral Sclerosis
