# Supplementary material for: Glutamatergic medications as adjunctive therapy for moderate to severe obsessive-compulsive disorder in adults: a systematic review and meta-analysis
Source: BMC Pharmacol Toxicol. 2021 Nov 4;22:69. doi: 10.1186/s40360-021-00534-6 (PMC8569963; doi:10.1186/s40360-021-00534-6)
Supplement: Supplementary file 1 — Additional file 1. [file 40360_2021_534_MOESM1_ESM.docx]

# Topic as in PICOT format

**P**: Adult patients with obsessive-compulsive disorder as defined by the Diagnostic and Statistical Manual of Mental Disorders (DSM–5), (irrespective of age, gender or race).

**I:** Using pharmaceutical agents that directly attenuate glutamatergic outﬂow as an adjunctive therapy.

**C:** Patients under treatment with SRIs. SRIs include clomipramine and the selective serotonin reuptake inhibitors (SSRIs).

**O**: Y-BOCS score reduction.

**T**: All clinical trials investigating glutamatergic agents (according the definitions in [I]), irrespective of randomization and blinding will be included.

# Search strategy

## Databases

### JOURNAL ARTICLES

## Medline

## Scopus

## Cochrane

***Keywords:***

P:

1. “Obsessive-compulsive disorder” [Title/Abstract]
2. Obsessive-compulsive[Title/Abstract]
3. OCD[Title/Abstract]
4. obsess* [Title/Abstract]
5. compulsi*[Title/Abstract]
6. “Obsessive-Compulsive Disorder” [Mesh]
7. #1 OR #2 OR #3 OR #4 OR #5 OR #6

I:

1. Modafinil[Title/Abstract]
2. glutamate*[Title/Abstract]
3. riluzol*[Title/Abstract]
4. memantin*[Title/Abstract]
5. ketamin*[Title/Abstract]
6. glycine[Title/Abstract]
7. sarcosin* [Title/Abstract]
8. topiramate[Title/Abstract]
9. lamotrigine[Title/Abstract]
10. N-acetylcysteine[Title/Abstract]
11. minocycline[Title/Abstract]
12. cycloserin*[Title/Abstract]
13. glutamatergic[Title/Abstract]
14. glutaminergic [Title/Abstract]
15. glutamin*[Title/Abstract]
16. NMDA[Title/Abstract]
17. AMPA [Title/Abstract]
18. kainate[Title/Abstract]
19. amantadin*[Title/Abstract]
20. atomoxetin* [Title/Abstract]
21. “N methyl D aspartate” [Title/Abstract]
22. dextromethorphan* [Title/Abstract]
23. NAC[Title/Abstract]
24. N acetyl cysteine*[Title/Abstract]
25. lanicemin*[Title/Abstract]
26. #8 OR #9 OR #10 OR #11 OR #12 OR #13 OR #14 OR #15 OR #16 OR #17 OR #18 OR #19 OR #20 OR #21 OR #22 OR #23 OR #24 OR #25 OR #26 OR #27 OR #28 OR #29 OR #30 OR #31 OR #32

#### T

1. randomized[Title/Abstract]
2. placebo[Title/Abstract]
3. randomly[Title/Abstract]
4. trial[Title/abstract]
5. randomized controlled trial[pt]
6. controlled clinical trial[pt]
7. clinical trial[pt]
8. #34 OR #35 OR #36 OR #37 OR #38 OR #39 OR #40 OR

#### Combined search

1. #7 AND #33 AND #41

## Restrictions

No language, filter or date restriction

# Specific issues:

- [PT] stands for publication type and is one of the filters available in PubMed
- [MeSH] only can be used in PubMed.
